# Supplementary material for: Multiscale Theoretical Study of Sulfur Dioxide (SO2) Adsorption in Metal–Organic Frameworks
Source: Molecules. 2023 Mar 31;28(7):3122. doi: 10.3390/molecules28073122 (PMC10096088; doi:10.3390/molecules28073122)
Supplement: Supplementary file 1 [file molecules-28-03122-s001.zip › molecules-2189848-supplementary.pdf]

Supporting Information for:

Multiscale Theoretical Study of Sulfur Dioxide (SO_2) Adsorption in Metal Organic Frameworks

Charalampos G. Livas [1], Dionysios Raptis [1], Emmanuel Tylianakis [1, 2] and George E. Froudakis [1, *]

[1] Department of Chemistry, University of Crete, Voutes Campus, GR-71003 Heraklion Crete, Greece
[2] Department of Materials Science and Technology, University of Crete, Voutes Campus, GR-71003 Heraklion Crete, Greece
* Correspondence: frudakis@uoc.gr

Ab-initio results.

In an attempt to identify both local minima and the energetically most favorable configuration of each system with the Sulfur dioxide molecule, various initial configurations for the dimer C_6H_5–X···SO_2 were considered and optimized. In Table S1 we present the systems under study along with their corresponding binding energy values. Figure S1 shows the global minimum configuration of all the complexes.

Table S1. [a] Sorted binding energies (kcal/mol) of the SO_2···C_6H_5–X systems under study, calculated at the RI-DSD-BLYP D3(BJ) /def2-TZVPP level of theory. All interaction energy values have been corrected for the Basis Set Superposition Error (BSSE) by the full counterpoise method [25]. [b] Percentage of binding energy enhancement with the introduction of the FG compared to benzene.

System	Binding Energy (kcal/mol)	Binding Energy Enhancement (%)
SO_2…C_6H_5-**PO_3H_2**	-10.1	146%
SO_2…C_6H_5-**OSO_3H**	-9.8	139%
SO_2…C_6H_5-**OPO_3H_2**	-9.3	127%
SO_2…C_6H_5-**CNH_2NOH**	-9.2	124%
SO_2…C_6H_5-**SO_3H**	-8.2	100%
SO_2…C_6H_5-**CH_2NH_2**	-8.1	98%
SO_2…C_6H_5-**NCH_2**	-7.7	88%
SO_2…C_6H_5-**NNH**	-7.6	85%
SO_2…C_6H_5-**$CONH_2$**	-7.5	83%
SO_2…C_6H_5-**SO_2NH_2**	-7.5	83%
SO_2…C_6H_5-**CHNOH**	-7.1	73%
SO_2…C_6H_5-**$OCONH_2$**	-7.1	73%
SO_2…C_6H_5-**$CONHNH_2$**	-7.0	71%
SO_2…C_6H_5-**$C(OH)_3$**	-7.0	71%
SO_2…C_6H_5-**$CONHCH_3$**	-6.7	63%
SO_2…C_6H_5-**SO_2CH_3**	-6.6	63%
SO_2…C_6H_5-**COOH**	-6.5	59%
SO_2…C_6H_5-**SOOH**	-6.3	54%
SO_2…C_6H_5-**OOH**	-6.1	49%

SO_2…C_6H_5-**NH_2**	-5.9	44%
SO_2…C_6H_5-**$NHCOCH_3$**	-5.9	44%
SO_2…C_6H_5-**CH_2OH**	-5.9	44%
SO_2…C_6H_5-**CHNH**	-5.6	37%
SO_2…C_6H_5-**CH_3**	-5.3	29%
SO_2…C_6H_5-**CH_2N_3**	-5.2	27%
SO_2…C_6H_5-**OC_2H_5**	-5.1	24%
SO_2…C_6H_5-**COOCHO**	-5.1	24%
SO_2…C_6H_5-**$CHCHNO_2$**	-4.9	20%
SO_2…C_6H_5-**COOCH3**	-4.9	20%
SO_2…C_6H_5-**SCN**	-4.7	15%
SO_2…C_6H_5-**SH**	-4.6	12%
SO_2…C_6H_5-**O_2CH_2**	-4.6	12%
SO_2…C_6H_5-**OH**	-4.5	10%
SO_2…C_6H_5-**SO_2Cl**	-4.5	10%
SO_2…C_6H_5-**CN**	-4.4	7%
SO_2…C_6H_5-**N_3**	-4.2	2%
SO_2…C_6H_5-**H**	-4.1	0%
SO_2…C_6H_5-**NCO**	-4.0	-2%
SO_2…C_6H_5-**NCS**	-4.0	-2%
SO_2…C_6H_5-**NC**	-3.9	-5%
SO_2…C_6H_5-**PH2**	-3.5	-15%
SO_2…C_6H_5-**F**	-3.5	-15%

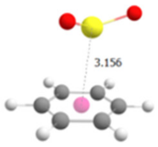

$SO_2...C_6H_5$-**H (ref)**

-4.1 kcal/mol

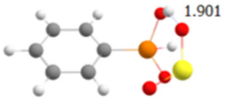

$SO_2...C_6H_5$-$\mathbf{PO_3H_2}$

-10.1 kcal/mol

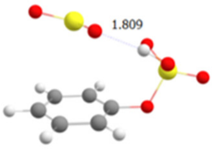

$SO_2...C_6H_5$-$\mathbf{OSO_3H}$

-9.8 kcal/mol

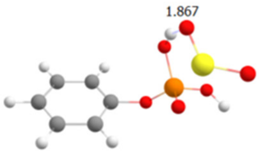

$SO_2...C_6H_5$-$\mathbf{OPO_3H_2}$

-9.3 kcal/mol

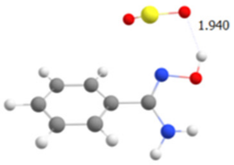

$SO_2...C_6H_5$-$\mathbf{CNH_2NOH}$

-9.2 kcal/mol

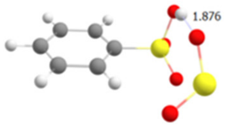

$SO_2...C_6H_5$-$\mathbf{SO_3H}$

-8.2 kcal/mol

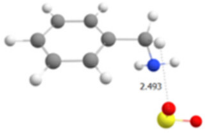

$SO_2...C_6H_5$-$\mathbf{CH_2NH_2}$

-8.1 kcal/mol

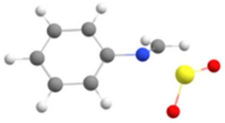

$SO_2...C_6H_5$-$\mathbf{NCH_2}$

-7.7 kcal/mol

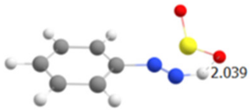

$SO_2...C_6H_5$-**NNH**

-7.6 kcal/mol

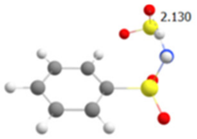

$SO_2...C_6H_5$-$\mathbf{SO_2NH_2}$

-7.5 kcal/mol

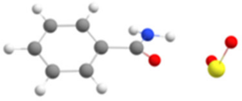

$SO_2...C_6H_5$-$\mathbf{CONH_2}$

-7.5 kcal/mol

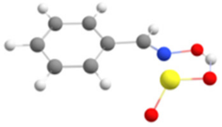

$SO_2...C_6H_5$-**CHNOH**

-7.1 kcal/mol

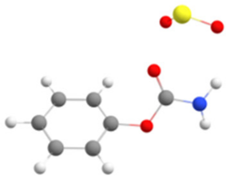

SO_2…C_6H_5-**$OCONH_2$**

-7.1 kcal/mol

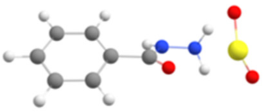

SO_2…C_6H_5-**$CONHNH_2$**

-7.0 kcal/mol

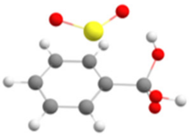

SO_2…C_6H_5-**$C(OH)_3$**

-7.0 kcal/mol

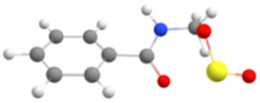

SO_2…C_6H_5-**$CONHCH_3$**

-6.7 kcal/mol

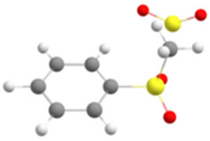

SO_2…C_6H_5-**SO_2CH_3**

-6.6 kcal/mol

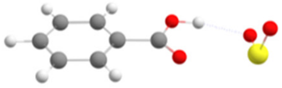

SO_2…C_6H_5-**COOH**

-6.5 kcal/mol

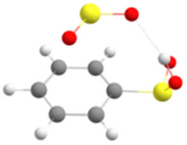

SO_2…C_6H_5-**SOOH**

-6.3 kcal/mol

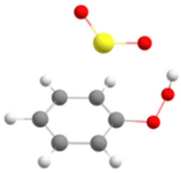

SO_2…C_6H_5-**OOH**

-6.1kcal/mol

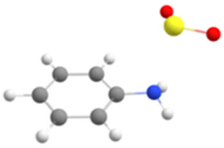

SO_2…C_6H_5-**NH_2**

-5.9 kcal/mol

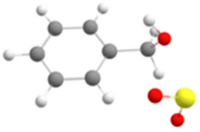

SO_2…C_6H_5-**CH_2OH**

-5.9 kcal/mol

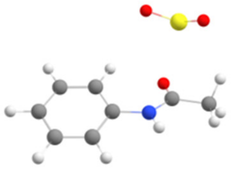

SO_2…C_6H_5-**$NHCOCH_3$**

-5.9 kcal/mol

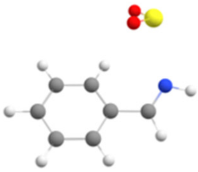

SO_2…C_6H_5-**CHNH**

-5.6 kcal/mol

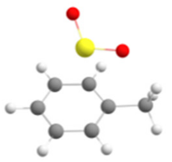

SO_2...C_6H_5-**CH_3**

-5.3 kcal/mol

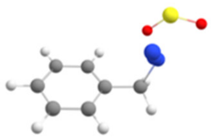

SO_2...C_6H_5-**CH_2N_3**

-5.2 kcal/mol

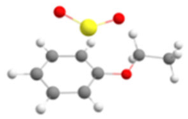

SO_2...C_6H_5-**OC_2H_5**

-5.1 kcal/mol

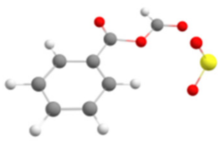

SO_2...C_6H_5-**COOCHO**

-5.1 kcal/mol

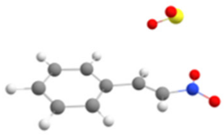

SO_2...C_6H_5-**$CHCHNO_2$**

-4.9 kcal/mol

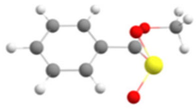

SO_2...C_6H_5-**$COOCH_3$**

-4.9 kcal/mol

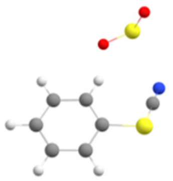

SO_2...C_6H_5-**SCN**

-4.7 kcal/mol

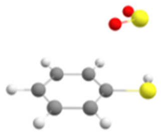

SO_2...C_6H_5-**SH**

-4.6 kcal/mol

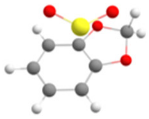

SO_2...C_6H_5-**O_2CH_2**

-4.6 kcal/mol

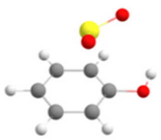

SO_2...C_6H_5-**OH**

-4.5 kcal/mol

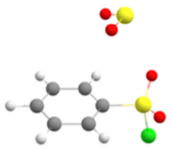

SO_2...C_6H_5-**SO_2Cl**

-4.5 kcal/mol

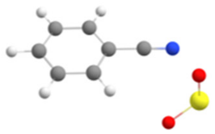

SO_2...C_6H_5-**CN**

-4.4 kcal/mol

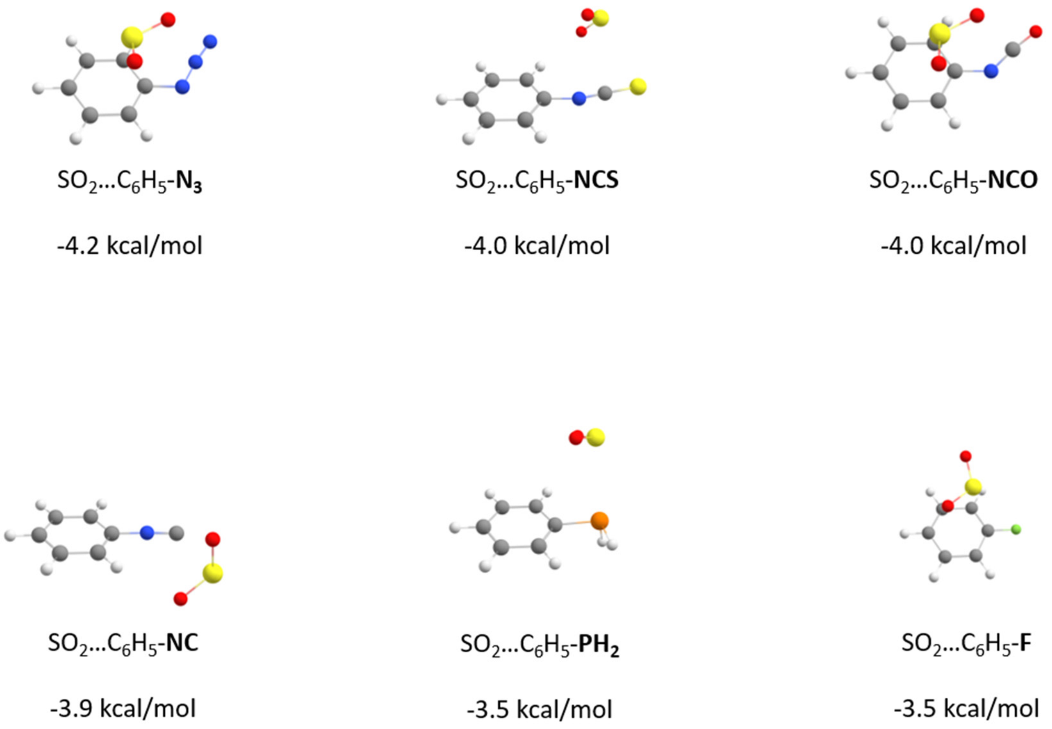

Figure S1. Global minima geometries and binding energy values (kcal/mol) of all the systems in this study.

Electron density difference plots were calculated at the RI-DSD-BLYP D3(BJ) /def2-TZVPP level of theory, as the difference of the electron density of the dimer minus the sum of the isolated monomers within the conformation of the dimer. The density of each monomer at the complex geometry was calculated in the presence of ghost basis functions of the other monomer. Densities were plotted with a contour value of 0.001 au by using gOpenMol [22] and are shown in Figure S2.

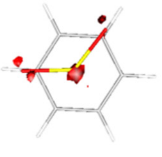

SO_2...C_6H_5-**H**

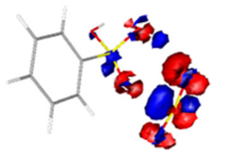

SO_2...C_6H_5-**PO_3H_2**

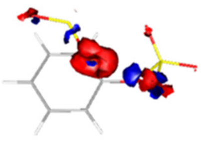

SO_2...C_6H_5-**OSO_3H**

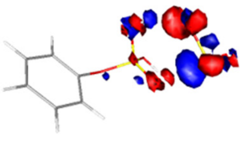

SO_2...C_6H_5-**OPO_3H_2**

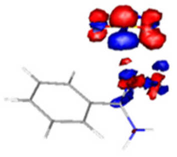

SO_2...C_6H_5-**CNH_2NOH**

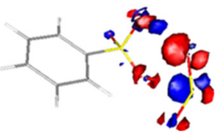

SO_2...C_6H_5-**SO_3H**

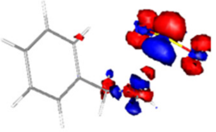

SO_2...C_6H_5-**CH_2NH_2**

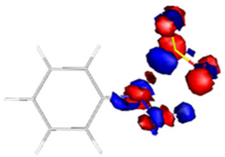

SO_2...C_6H_5-**NNH**

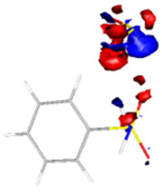

SO_2...C_6H_5-**SO_2NH_2**

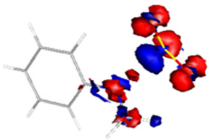

SO_2...C_6H_5-**NCH_2**

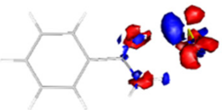

SO_2...C_6H_5-**$CONH_2$**

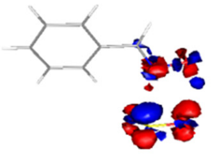

SO_2...C_6H_5-**CHNOH**

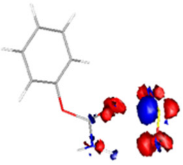

SO_2...C_6H_5-**$OCONH_2$**

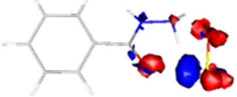

SO_2...C_6H_5-**$CONHNH_2$**

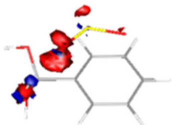

SO_2...C_6H_5-**$C(OH)_3$**

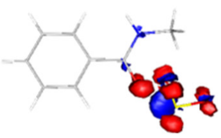

SO_2...C_6H_5-**$CONHCH_3$**

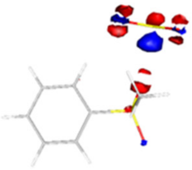

SO_2...C_6H_5-**SO_2CH_3**

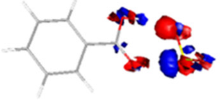

SO_2...C_6H_5-**COOH**

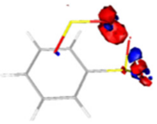

SO_2...C_6H_5-**SOOH**

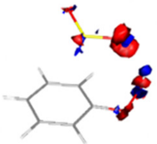

SO_2...C_6H_5-**OOH**

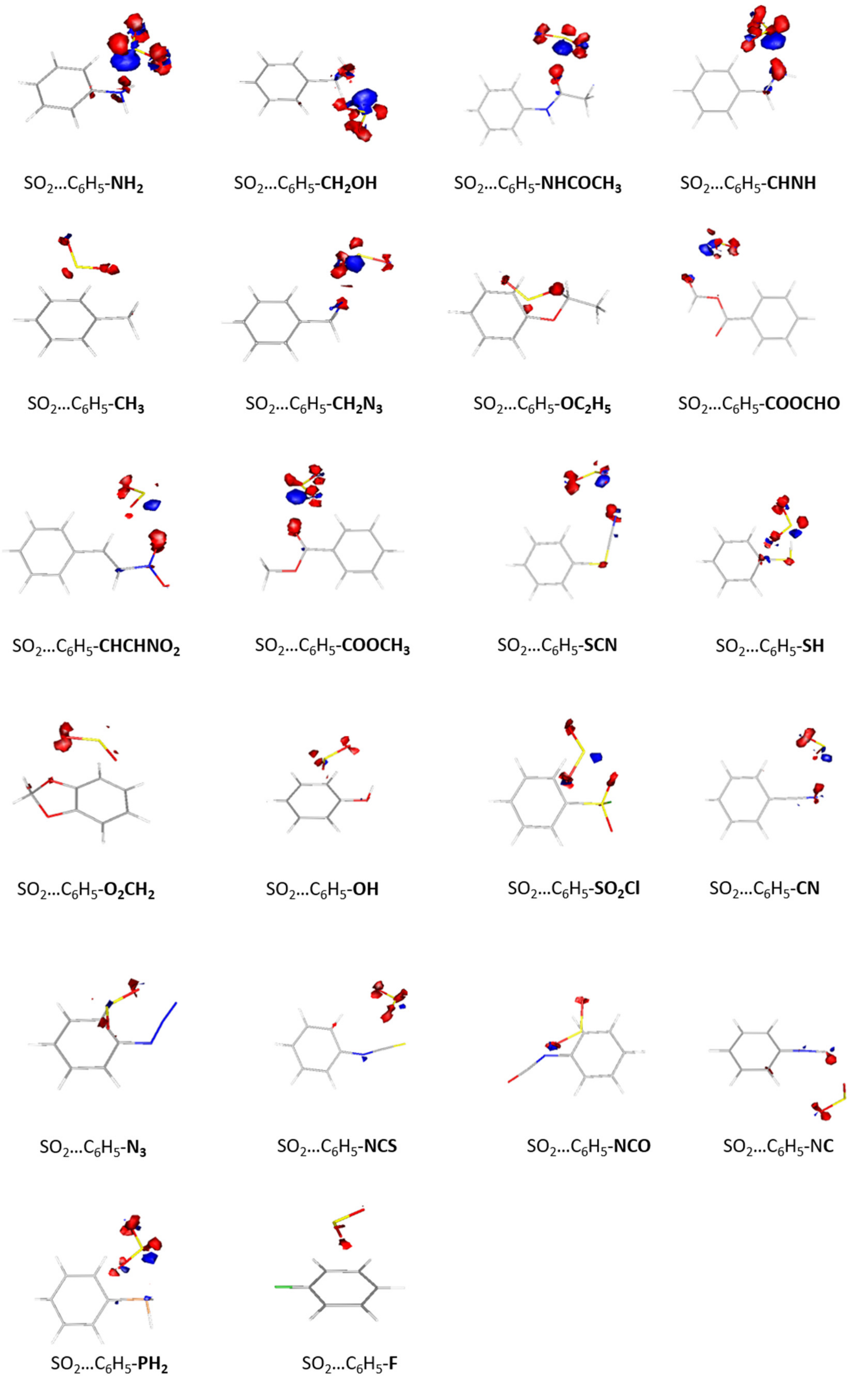

Figure S2. Electron-density redistribution plots of the optimized geometries of the $SO_2\cdots C_6H_5$–X complexes. With red and blue the regions that gain and lose electron density upon the formation of the complex, respectively.

Ab-initio derived interatomic potential fitting

In order to obtain the QM points, i.e., the interaction energies between the hydrogen molecule and the C_6H_5-X moiety, a rigid scan of 40 single-point calculations was performed at the RI-DSD-BLYP D3(BJ)/def2TZVPP level for each functional group selected. During these calculations, we held fixed the position of the functionalized benzene at the global (or local) minimum energy configuration of the dimer and sampled a selected distance, moving hydrogen from 5.5 to 1.5 Å towards the functional group position. Functionalized linkers were represented with functionalized benzenes. Since the FGs contain many different atom types whose parameters *ε* and *σ* need to be fitted simultaneously, we examined also the local minima. For each different local minimum, a different FG atom plays the dominant role in the interaction energy for the specific site. By fitting the specific curves for all different atoms using an in-home parametrization algorithm, we get the parameter values for all atoms of the FG. All parameters were mixed using the Lorentz-Berthelot mixing rules [31,32].

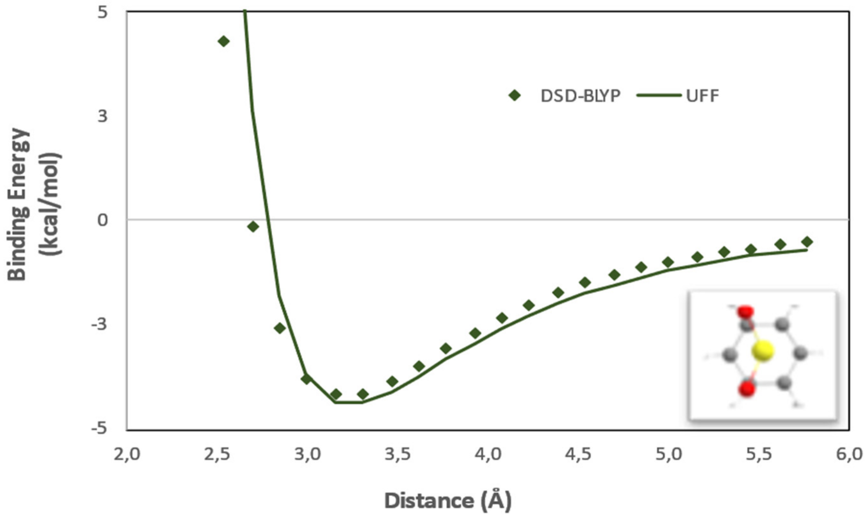

Figure S3. Fitting of the (ε, σ) parameters of the UFF [30] potential on the QM data obtained from the ab-initio scan of SO_2 over benzene

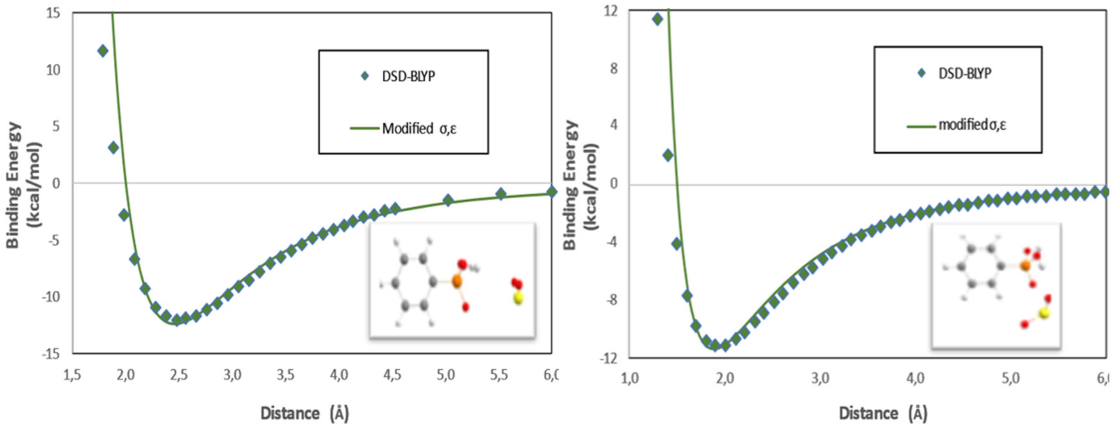

Figure S4. Fitting of the (ε, σ) parameters for the SO···C_6H_5–PO_3H_2 interaction in two different directions. With rhombus, the QM scans around the global minimum conformation of the dimer.

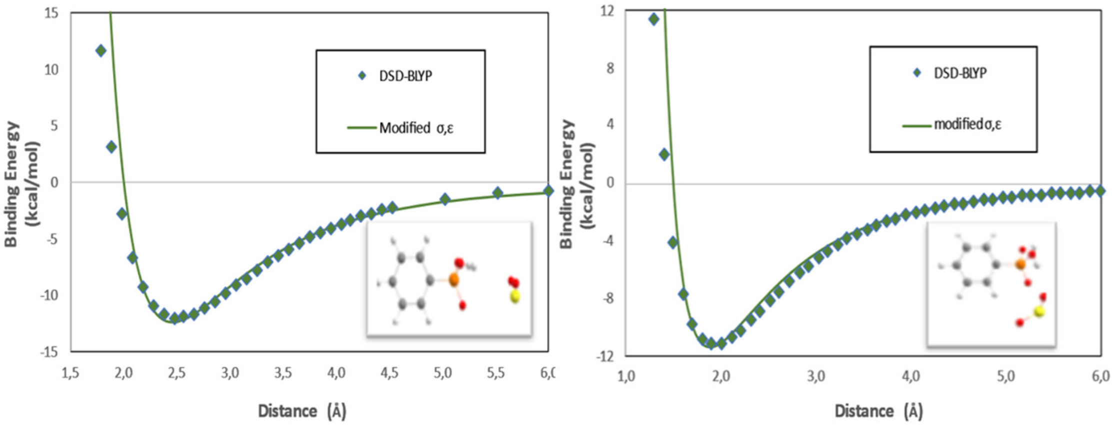

(a) (b)

Figure S5. Fitting of the (ε, σ) parameters for the SO···C_6H_5–CNH_2NOH interaction in two different directions. With rhombus, the QM scans around the global minimum conformation of the dimer.

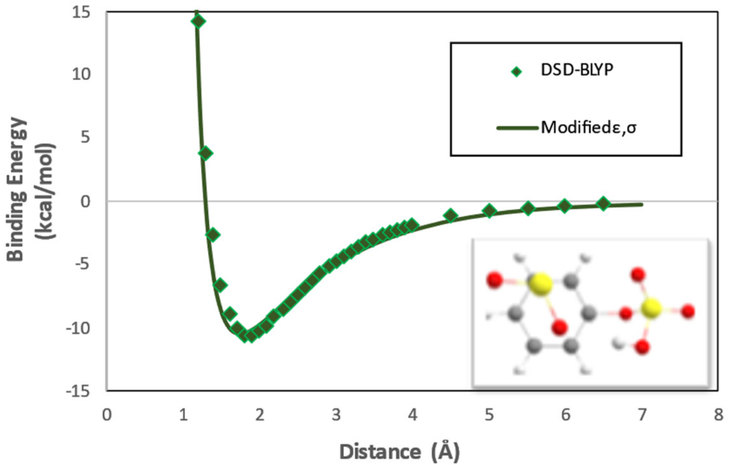

Figure S6. Fitting of the (ε, σ) parameters for the SO_2···C_6H_5–OSO_3H interaction. With rhombus, the QM scans around the global minimum conformation of the dimer.

The pronounced difference between UFF [30] and the ab-initio curve verifies the importance of the described fitting procedure and indicates the risk of employing classical FFs without first checking their validity on the system under study.

References

22. Laaksonen L. A graphics program for the analysis and display of molecular dynamics trajectories. J Mol Graph 1992;10:33–4. https://doi.org/https://doi.org/10.1016/0263-7855(92)80007-Z.

25. Boys SF, Bernardi F. The calculation of small molecular interactions by the differences of separate total energies. Some procedures with reduced errors. Mol Phys 1970;19:553–

66. https://doi.org/10.1080/00268977000101561.

30. Rappe AK, Casewit CJ, Colwell KS, Goddard WA, Skiff WM. UFF, a full periodic table force field for molecular mechanics and molecular dynamics simulations. J Am Chem Soc 1992;114:10024–35. https://doi.org/10.1021/ja00051a040.

31. Berthelot D. Sur le mélange des gaz. Comptes Rendus Hebd Des Séances l'Académie Des Sci 1898;126:1703–855.

32. Lorentz HA. Ueber die Anwendung des Satzes vom Virial in der kinetischen Theorie der Gase. Ann Phys 1881;248:127–36. https://doi.org/10.1002/andp.18812480110.
